# Skyrmionium – high velocity without the skyrmion Hall effect

**DOI:** 10.1038/s41598-018-34934-2

**Published:** 2018-11-16

**Authors:** Alexander G. Kolesnikov, Maksim E. Stebliy, Alexander S. Samardak, Alexey V. Ognev

**Affiliations:** 1 School of Natural Sciences, Far Eastern Federal University, Vladivostok, Russia; 2 National Research South Ural State University, Chelyabinsk, Russia

## Abstract

The lateral motion of a magnetic skyrmion, arising because of the skyrmion Hall effect, imposes a number of restrictions on the use of this spin state in the racetrack memory. A skyrmionium is a more promising spin texture for memory applications, since it has zero total topological charge and propagates strictly along a nanotrack. Here, the stability of the skyrmionium, as well as the dependence of its size on the magnetic parameters, such as the Dzyaloshinskii–Moriya interaction and perpendicular magnetic anisotropy, are studied by means of micromagnetic simulations. We propose an advanced method for the skyrmionium nucleation due to a local enhancement of the spin Hall effect. The stability of the skyrmionium being in motion under the action of the spin polarized current is analyzed.

## Introduction

A skyrmion is a stable magnetic configuration having a small size (10–100 nm)^1–3^, as well as a high propagation velocity (~100 m/s) under the action of low-density currents (~1 × 10^9^ А/м^2^)^4–7^. Due to these features, the skyrmion can be used for a new type of magnetic memory called the racetrack memory^8,9^. However, the presence of the skyrmion Hall effect (SkHE)^10–12^, arising due to the topological properties of the skyrmion, limits its use in narrow tracks. Under the influence of the transverse Magnus force, the skyrmion deviates from the longitudinal motion along a nanotrack, which can lead to annihilation of the skyrmion at a track’s edge^13^. To solve this problem, several methods have been proposed that can prevent touching the edges and keep the skyrmion inside the track: changing the track’s profile through the creation of additional borders (kerbed track)^14^, local modification of magnetic properties of a nanotrack, such as perpendicular magnetic anisotropy (PMA)^6,15^, the interfacial Dzyaloshinskii–Moriya interaction (iDMI)^16^ or damping constant^17,18^. All these approaches allow creating a potential barrier that prevents the skyrmion’s deviation. Another method is based on the topological compensation of the Magnus effect. Since the direction of the skyrmion’s deflection depends on the sign of the topological charge (Q), then spin states that have a compensated total topological charge (Q = 0) must not deviate. Magnetic configurations consisting of two antiferromagnetically coupled skyrmions with opposite topological charges have been theoretically investigated in ferrimagnets^19–21^, antiferromagnets^22–27^, and synthetic antiferromagnets^28–30^. However, these configurations are difficult to realize and to investigate experimentally, because of the complexity of visualization of the antiferromagnetic domain structure. In ferromagnetic media, a skyrmion-like state with zero topological charge, which is called skyrmionium^31–35^, can be stabilized. For the first time a skyrmionium was described in a theoretical work and it was named as “2π-vortex”^36^. In the later papers the skyrmionium state was found by modeling or experimentally and called as “donut skyrmion”^37^, “2π-skyrmion”^38^ and “target skyrmion”^39^. A skyrmionium is a skyrmion surrounded by an annular domain wall with the opposite Q. This spin structure is elastically coupled due to magnetostatic repulsion between the skyrmion and the surrounding domain wall.

In this paper, we explore the possibility of using the skyrmionium to implement the racetrack memory. We propose a new method for the nucleation of the skyrmionium based on a locally enhanced spin-orbit torque (SOT) effect. Conditions for the formation of the skyrmionium are determined by means of micromagnetic simulations. The influence of the magnetic parameters on the size and stability of the skyrmionium is analyzed. The skyrmionium motion under the action of the spin currents is studied.

## Skyrmionium Nucleation

**Figure 1:**
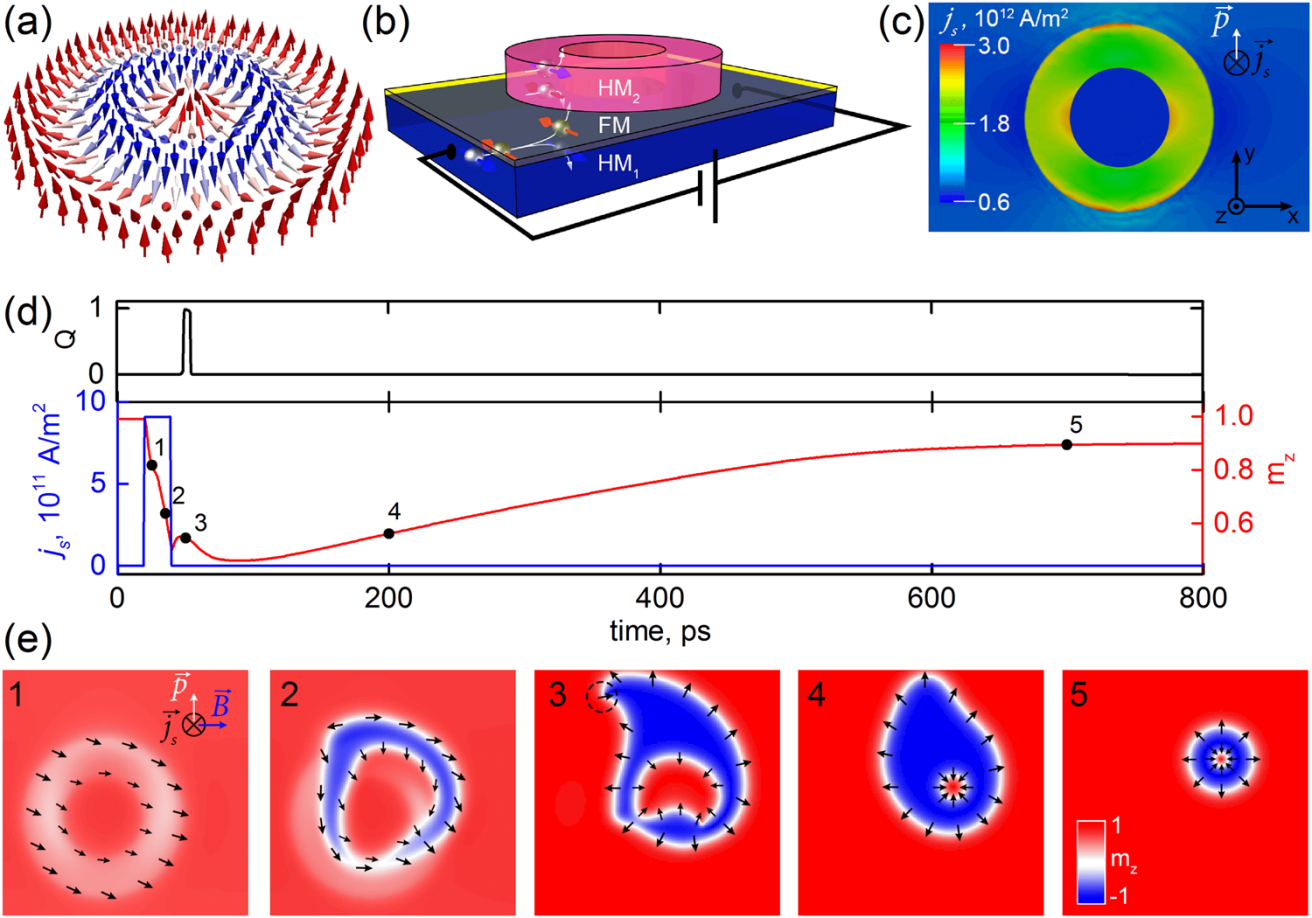
Nucleation of the skyrmionium with the help of the spin-orbit torque. (**a**) Spin configuration of the skyrmionium. (**b**) Scheme of the spin Hall effect in the *HM*_1_*/FM/HM*_2_ structure. (**c**) Distribution map of the spin current density (*j*_*s*_) injected into the ferromagnetic layer. (**d**) The time dependence of the integral density of the spin current *j*_*s*_ = *f(t)* passed through the ferromagnetic layer (blue curve), the corresponding changes in the perpendicular magnetization component *m*_*z*_ = *f(t)* (red curve) and the magnitude of the total topological charge *Q* = *f(t)* (black line). (**e**) Visualization of the processes of changing the magnetization distribution in the ferromagnetic layer for the selected time instants (1–5), indicated on the *m*_*z*_ = *f(t)* graph.

To apply a skyrmionium in the racetrack memory, it is necessary to be able to control the nucleation and annihilation processes of this quasi-particle inside nanotracks. A magnetic skyrmionium is a composite structure consisting of a skyrmion (skyrmionium core) surrounded by an annular domain wall with an opposite topological charge (skyrmionium shell). The scheme of the skyrmionium is shown in Fig. 1(a). For nucleation of skyrmions and skyrmioniums, the optical means can be used^40–44^. However, the current-induced nucleation is technically simple for realization and compatible with the CMOS technology. In previous studies it was demonstrated that the skyrmionium can be generated inside a nanodisk using the spin-transfer torque (STT) by locally passing the spin polarized current ($\overrightarrow{p}||-\,\overrightarrow{z}$) perpendicularly to the nanodisk plane^33^. However, the experimental implementation of this method is quite problematic, because of the fabricating complexity of a circular shape nanocontact. In this case, the magnetostatic interaction between the skyrmionium and the polarizing spin filter will prevent the displacement of the skyrmionium from the contact. This will lead to an increase in the critical current required for the skyrmionium movement.

We propose to use the SOT for the skyrmionium nucleation^45–47^. To realize the possibility of nucleation of domains of a given shape, for example, annular, it is necessary to locally change the SOT magnitude. In a “ferromagnet/heavy metal” system (*FM/HM*) the SOT magnitude depends on parameters of *HM* (thickness, crystal structure) being in contact with *FM*^48–50^. To strengthen the SOT, one can deposit a heavy metal with an opposite spin Hall angle (*θ*_*SH*1_ > *0*, *θ*_*SH*2_ < *0*) on the second interface to realize the *HM*_*1*_*/FM/HM*_2_ structure^51^. As shown in^51^, the total value of *θ*_*SH*_ increases from 0.1 for a Pt/Co bilayer to 0.34 for a Pt/Co/Ta system. Through the patterning of *HM*_*2*_, one can specify regions of a given geometry with the large value of *θ*_*SH*_. To generate the skyrmionium, we need to switch the magnetization of the skyrmionium shell in the opposite direction relatively to the skyrmionium core. For this purpose we used a nanostructure consisting of a nanoring made of *HM*_*2*_ located on the top of a *HM*_1_*/FM* film, Fig. 1(b).

In the proposed Pt/Co/Ta structure the bottom Pt/Co interface has the main contribution to the net iDMI value, because it has been experimentally shown that Ta does not induce a significant iDMI (it is about 20 times smaller compare to Pt/Co interface)^52^. We have done preliminary numerical simulations considering the local enhancement of iDMI in the area under the Ta circle, Fig. S1(a) in the Supplementary file. Using this iDMI distribution we have modelled the skyrmionium nucleation processes. The calculated results are in a good agreement with the result for a case of the uniform iDMI, Fig. S1(b) in the Supplementary file. Thus, the assumption on the uniformity of iDMI in the proposed structure is correct. The larger variations of iDMI (higher than 10%) will affect on the skyrmionium stability and the critical current of its nucleation.

We calculated the distribution of the charge current density ${j}_{x}^{ijk}$, where *ijk* – coordinates of an elemental cell, passing through the film and nanoring, taking into account the resistivity of metals and the nanostructure’s size. The thickness of the buffer layer *HM*_1_*(Pt)* and the nanoring *HM*_2_*(Ta)* was 4 nm. The nanoring *HM*_2_*(Ta)* had the external and internal diameters of 100 and 50 nm, correspondingly. Using the distribution of ${j}_{x}^{ijk}$ and the spin Hall effect (SHE) in the Pt film (${\theta }_{SH}^{film}=0.1$) and in the Ta nanoring (${\theta }_{SH}^{ring}=0.24$), for each elemental cell we computed the spin current (${\overrightarrow{j}}_{s}||-\,z$) with the polarization $\overrightarrow{p}||\overrightarrow{y}$, which was generated perpendicularly to the plane of the FM layer accordingly to the equation ${j}_{s}^{ij}={j}_{x}^{ijk}\cdot {\theta }_{SH}^{film}+{j}_{x}^{ijk}\cdot {\theta }_{SH}^{ring}$. The derived distribution map of ${j}_{x}^{ijk}$ is shown in Fig. 1(c).

Using the spin current distribution map, we carried out simulations of the skyrmionium nucleation processes in a nanotrack with the size of 1000 × 400 nm^2^. These calculations were done in the micromagnetic modeling program MuMax^3^ ^53^. The thickness of the ferromagnetic layer was 0.6 nm, the size of the elemental cell was 1.0 × 1.0 × 0.6 nm^3^. Magnetic parameters of Co were chosen accordingly to the literature data^8,51,54^: saturation magnetization *M*_*s*_ = 580 kA/m; the exchange interaction constant *A* = 15 pJ/m; the PMA energy *K* = 1.5 MJ/m^3^; the iDMI energy *D* = 5 mJ/m^2^; the Gilbert damping constant *α* = 0.3. In order to avoid the formation of magnetostatic charges at the edges of the simulated area, the simulation was performed taking into account the periodic boundary conditions along the coordinate axes *Ox* and *Oy*.

Let us consider the nucleation processes of the skyrmionium under the action of a current pulse. Initially, the ferromagnetic layer was in a saturation state along the *Oz* axis. To realize the local magnetization reversal with the help of the SOT, we have to pass through the structure a charge current pulse with the amplitude *j*_*x*_ = *−*8.6 × 10^12^ A/m^2^ and duration of 20 ps. Due to the SHE the spin current pulse with a maximum amplitude passes under the nanoring through the *FM* layer, Fig. 1(c). The average value of the spin-current amplitude under the nanoring is *j*_*s*_ = 9.1 × 10^11^A/m^2^ (see the blue curve in Fig. 1(d)). Simultaneously, a pulse of a uniform external magnetic field *B*_*x*_ = 0.6 T was applied to the structure along the *Ox* axis. Under the influence of the current and magnetic field, a decrease in *m*_*z*_ magnetization component is observed and an annular shape domain is formed (Fig. 1(e), instants 1 and 2). In this case, the spin current torque acting on the domain walls displaces the nucleated domain. To prevent further growth and displacement of the nanoring domain, the external excitation was stopped on 40 ps (*j*_*s*_ = 0 A/m^2^ and *B*_*x*_ = 0 T).

Initially, the domain wall is not homochiral (Fig. 1(e), instant 2), because the magnetization in the domain wall was directed mainly along the effective field. After switching off the external excitation, self-organization of the magnetization configuration takes place. In thin *FM* layers with the high PMA and positive iDMI, Néel’s domain walls with left-handed chirality are stabilized^55,56^. Moreover, a full magnetization turn inside the domain wall occurs more rapidly in the central domain, while there is an area with right-handed chirality in the outer domain wall, marked in the instant 3 of Fig. 1(e) by a dashed line. Thus, the skyrmion is stabilized at 50 ps. The moment of the skyrmion’s formation is reflected by an abrupt jump in the topological charge from 0 to 1 as seen in the graph *Q* = *f(t)*, Fig. 1(d).

After this, the area with right-handed chirality in the outer domain wall annihilates, the wall acquires left-handed chirality. Then the topological charge of the external domain wall will be −1, and the total topological charge will be zero again. The resulting skyrmionium has an asymmetric shape and a large size, Fig. 1(e), instant 4. Within 500 ps, a decrease in the size of the skyrmionium occurs with the following transition to the equilibrium state, Fig. 1(e), instant 5. As the size of the skyrmionium decreases, the value of *m*_*z*_ in the considered region increases, Fig. 1(d). The complete process of the skyrmionium’s nucleation and stabilization for these magnetic parameters lasts about 700 ps. The skyrmionium spin dynamics corresponding to Fig. 1(e) is demonstrated in the Supplementary Movie.

In order to analyze the effect of the Gilbert damping on the nucleation process of a skyrmionium, the micromagnetic simulations with different values of α were performed. Since the pulsed excitation by the external magnet field and spin current of 20-ps duration is used for the skyrmionium nucleation, then with the increasing α the current density required for the nucleation of a domain of the circular shape (nanoring domain) also increases, Fig. S2(b) in the Supplementary file. If *α* changes from 0.2 to 1.0 the nucleation process keeps constant. Practically the same shape of the *m*_*z*_ = *f(t)* curves supports this suggestion, Fig. S2(a) in the Supplementary file. The only change is the rising time required for stabilization of the homochiral domain walls and relaxation of a domain to the equilibrium size. At *α* = 0.1 during the excitation time of 20 ps in spite of a nanoring domain the magnetic bubbles are also nucleated. Because of the small size of these bubbles (less than 10 nm), they are unstable and annihilate inducing the excitation of spin waves affecting on the shape of domain walls and on the transfer time of the skyrmionium into the stable state. To prevent any parasitic effects at small values of α, time of the pulsed excitation has to be decreased in this case. These results demonstrate that magnetic materials with the small Gilbert damping constant (*α* ≤ 0.1) are not good candidates for skyrmionium media.

## Topological Stability of Skyrmionium

To implement the skyrmionium-driven racetrack memory, it is necessary to know a range of magnetic parameters under which this topological state will remain stable. In the *HM*_1_*/FM/HM*_2_ systems, a replacement of heavy metals contacting with *FM* or a change in the thickness of the layers will primarily affect such parameters of the ferromagnetic layer as the PMA energy and iDMI^57–59^. Changing of these magnetic parameters will influence the skyrmionium size and, consequently, the density of information recording in magnetic memory.

**Figure 2:**
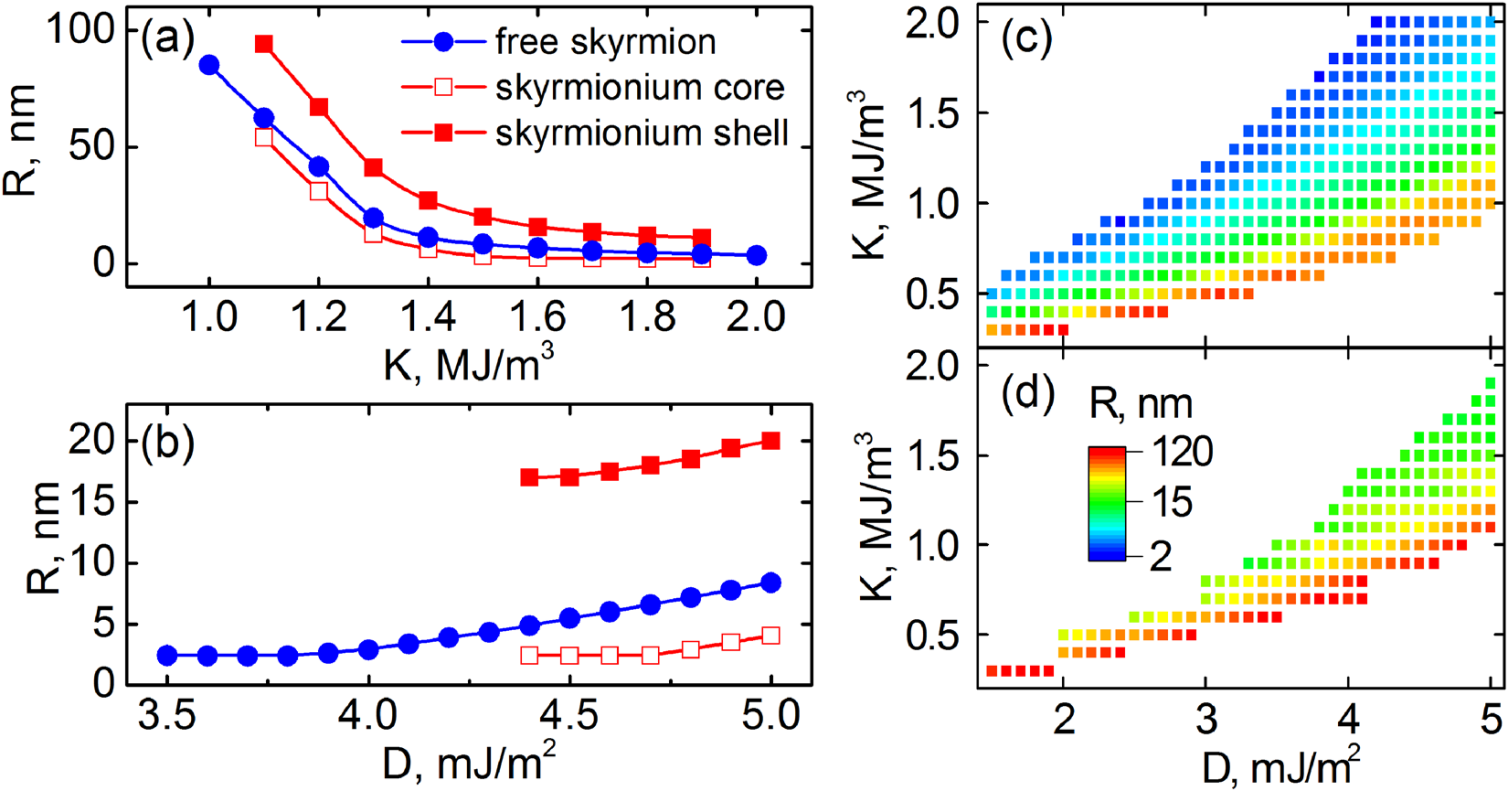
Dependences of the radius of the free skyrmion, skyrmionium’s core and shell on (**a**) the PMA magnitude for the fixed *D* = 5 mJ/m^2^, on (**b**) the iDMI constant for the fixed *K* = 1.5 MJ/m^3^. Diagrams of the radii of the skyrmion (**c**) and the skyrmionium (**d**) in the *D* and *K* coordinates.

To find the skyrmionium stability range, the anisotropy energy *K* and the interfacial Dzyaloshinsky-Moriya interaction constant *D* were varied in the range from 0.3 to 2.0 MJ/m^3^ and from 1.5 to 5.0 mJ/m^2^, correspondingly. For comparison, the stability region of a free skyrmion was also found. It is known that as the PMA energy increases, the skyrmion radius decreases^2^, (blue curve in Fig. 2(a)). In this case, the magnetostatic interaction between the homochiral walls in the skyrmionium core and shell leads to compression of the skyrmionium core in comparison with the free skyrmion (red curve with empty symbols in Fig. 2(a)).

With an increase in the iDMI energy density for the fixed PMA, the skyrmionium radius or its shell width increases also as in the case of the free skyrmion^60^, Fig. 2(b). Thus, the magnetic parameters *K* and *D* allow changing the skyrmionium size in a wide range. An increase in the recording density by decreasing the skyrmionium size is possible due to an increase in the PMA energy and a decrease in the iDMI constant. In this case, there is a certain range of magnetic parameters for which the skyrmionium is stable. The region of the stable skyrmionium depending on the *K* and *D* values is shown in Fig. 2(d), the color in this diagram corresponds to the equilibrium radius of the skyrmionium at these parameters. For comparison, Fig. 2(c) shows a similar diagram for a stable skyrmion. It was found that the stability region for the skyrmionium is smaller than for the skyrmion. If in the case of the skyrmion the stability region is bounded from above by a single-domain state, then at a high *K* or a weak *D* the structure will be in the saturation state. At a low *K* and a high *D*, a strip domain structure will be formed. The decrease of the skyrmionium stability region is due to the fact that the skyrmionium core contracts and annihilates at a lower anisotropy than the free skyrmion, because of its magnetostatic interaction with the outer shell.

Thus, magnetic parameters at which it is possible to implement a stable skyrmionium in the trilayer structure were found. In the range of magnetic parameters considered, the skyrmionium radius can be varied from 12 to 120 nm by varying the PMA energy and iDMI constant. Below we consider the stability of the skyrmionium being in motion under the action of the spin polarized current.

## Spin Current Driven Motion Of Skyrmionium

Skyrmionium can be moved inside a nanotrack by spin waves^61,62^, magnetic field gradient^31,33^ and spin-polarized current^32,34,63^. In this section, we demonstrate the spin current-dependent dynamics of a skyrmionium in comparison with a skyrmion and analyze the skyrmionium stability under the current action.

**Figure 3:**
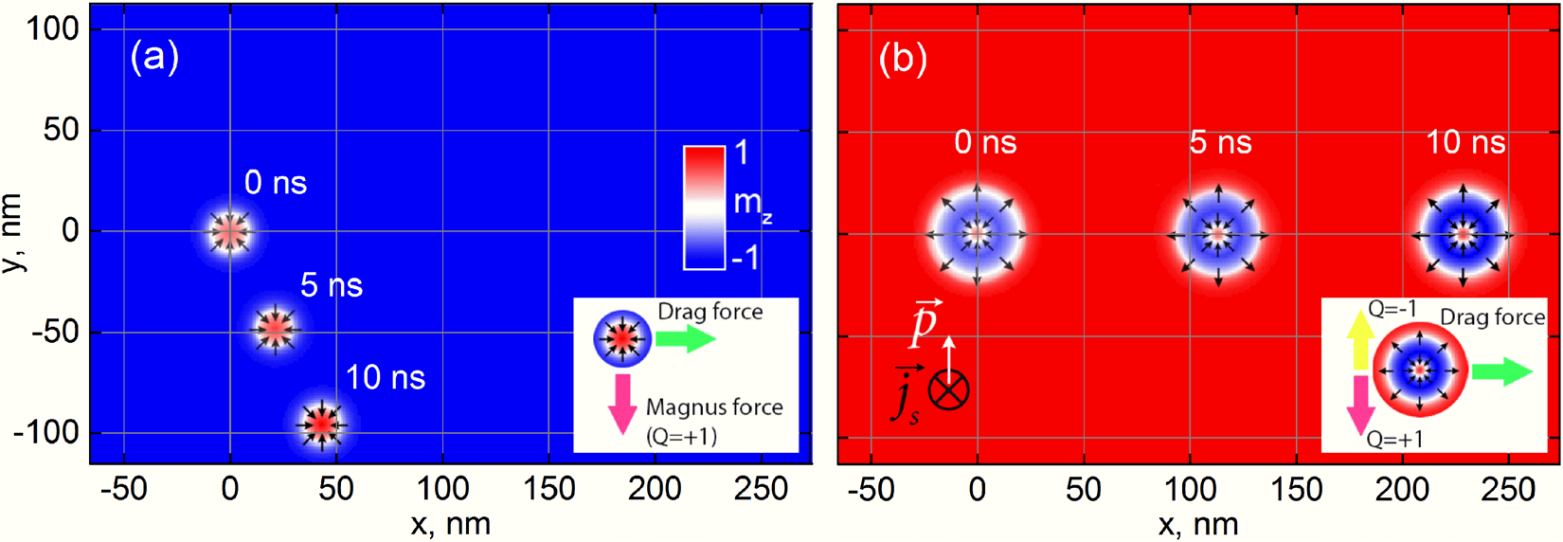
Simulation of the motion of the skyrmion (**a**) and the skyrmionium (**b**) under the current action (*j*_*s*_ = 1 × 10^10^ A/m^2^). Insets schematically reflect the forces acting on corresponding topological textures in motion.

As it was shown above, to nucleate a skyrmionium the spin current density and the applied magnetic field have to be ~2 × 10^12^ A/m^2^ and 0.6 T, correspondingly. The significantly smaller current density (~1 × 10^10^ A/m^2^) is needed for the skyrmionium movement without any external magnetic field. To move the skyrmionium towards the direction $+\overrightarrow{x}$ the spin current has to be applied perpendicularly to the FM layer (along $-\overrightarrow{z}$) with the polarization along $+\overrightarrow{y}$. Under the action of the same current, a skyrmion has non-zero components of velocity −*V*_*x*_ and *V*_*y*_ forcing it to move to the nanotrack edge, Fig. 3(a). The reason for that is the skyrmionic Hall effect resulting in the appearance of two mutually perpendicular forces acting on the skyrmion: the drag force is causing in the skyrmion motion along $+\overrightarrow{x}$ and the deflecting Magnus force is directed along $-\overrightarrow{y}$ at *Q* = +1 (see the inset of Fig. 3(a)). The skyrmionium core and shell have opposite topological charges +1 and −1, correspondingly. Thus, in addition to the drag force also directed along the nanotrack, there will be two Magnus forces acting on the skyrmionium, which are oriented down for the core and up for the shell as shown in the inset of Fig. 3(b). If the core and the shell are elastically coupled, then the Magnus forces will cancel each other allowing the skyrmionium to move strictly along the nanotrack.

To simulate the motion of both spin textures under the current action, we used the same magnetic parameters as for the modelling of the skyrmionium nucleation: *M*_*s*_ = 580 kA/m; *A* = 15 pJ/m; *K* = 1.5 MJ/m^3^; *D* = 5 mJ/m^2^; *α* = 0.3. At these parameters the free skyrmion moves at angle *θ*_*SkH*_ = *tan*^*−1*^(*V*_*y*_/*V*_*x*_) = 65° relatively to the nanotrack’s main axis. This angle is called the skyrmion Hall effect angle. Results of the micromagnetic simulations are in a good agreement with the analytical calculations using the formula *θ*_*SkH*_ *≈* *tan*^*−1*^*(*2*Δ/αR)* = 68°, where ${\rm{\Delta }}=\sqrt{\frac{A}{{K}_{eff}}}=3.4\,nm$ is the domain wall width, *K*_*eff*_ = *K-2πM*_*s*_^*2*^ is the effective anisotropy energy, *R* = 9 nm is the skyrmion radius (Fig. 2(a))^5^.

**Figure 4:**
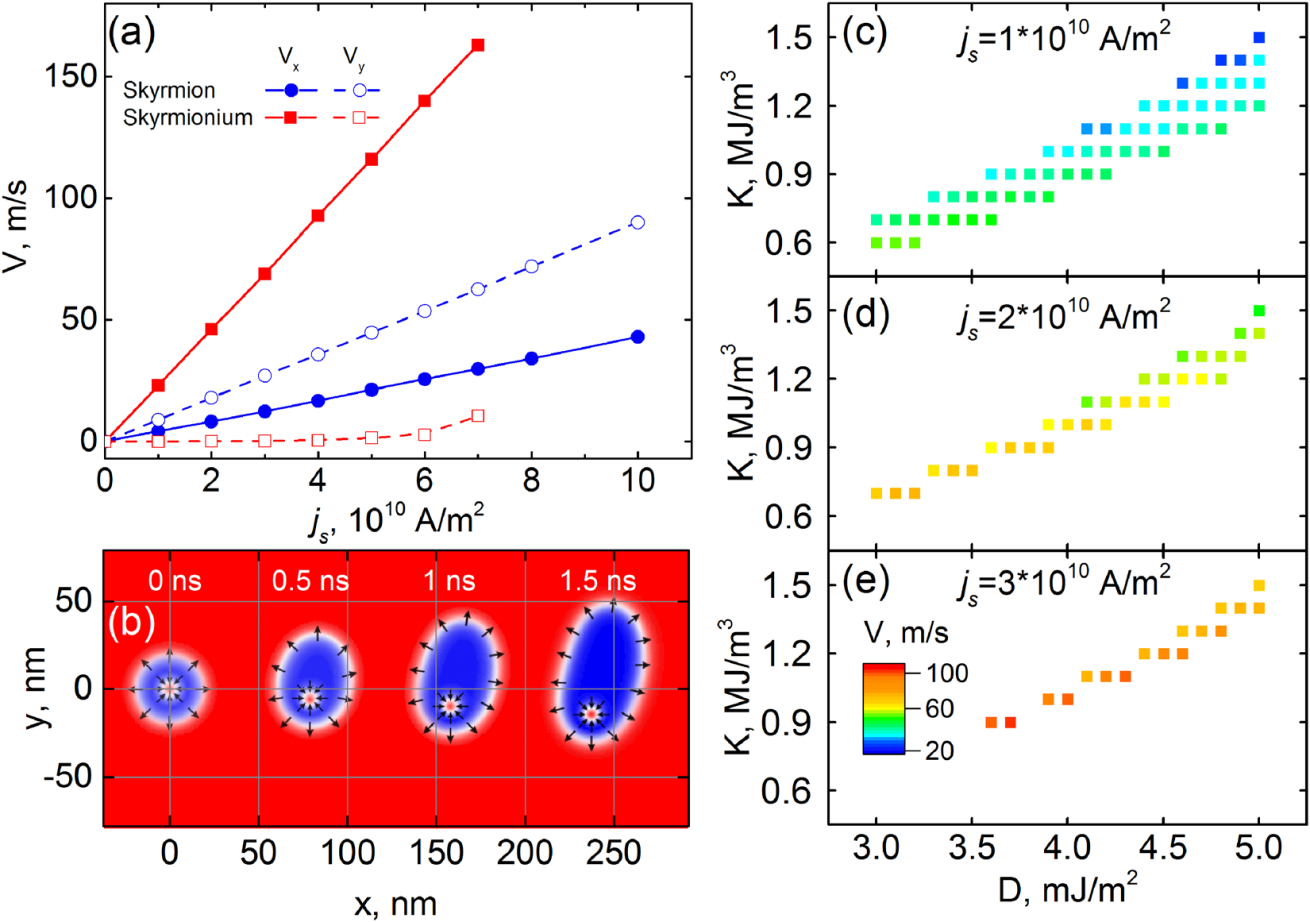
(**a**) Dependences of *V*_*x*_ and *V*_*y*_ on the spin current density for the skyrmion and the skyrmionium. (**b**) Skyrmionium’s deformation in motion under the action of the spin current *j*_*s*_ = 7 × 10^10^ A/m^2^. Diagrams of the absolute velocity *V* of the skyrmionium in coordinates *D* and *K* for various current densities: (**c**) 1 × 10^10^ A/m^2^, (**d**) 2 × 10^10^ A/m^2^, (**e**) 3 × 10^10^ A/m^2^.

As it was defined from the simulations, with the increase of the applied current from 1 to 10 × 10^10^ A/m^2^ the velocity’s components *V*_*x*_ and *V*_*y*_ grow linearly (Fig. 4(a)) resulting in the constant value of *θ*_*SkH*_. In the case of the skyrmionium motion, there are two transverse forces applied to different points: one force drags the core down, while the second one drags the shell up. These transverse forces will try to stretch the skyrmionium shell. At the current magnitude *j*_*s*_ ≤ 5 × 10^10^ A/m^2^ the skyrmionium shell does not deform and the core does not shift relatively to the nanotrack’s main axis. In other words, we observe the straightforward motion of the skyrmionium towards the nanotrack as shown in Fig. 3(b). At that *θ*_*SkH*_ = 0 and the longitudinal velocity *V*_*x*_ is in 5.5 times larger than for the skyrmion, Fig. 4(a).

The increase of the current density up to *j*_*s*_ > 5 × 10^10^ A/m^2^ leads to the stretching of the skyrmionium shell and the core moves in the transverse direction, Fig. 4(b). Even in this case the coupling between the core and the shell is significantly slowing down the transverse velocity *V*_*y*_ if to compare with the free skyrmion. At *j*_*s*_ = 7 × 10^10^ A/m^2^ the transverse velocity of the skyrmionium core is just 10 m/s (*θ*_*SkH*_ *≈* 4°), while for the free skyrmion *V*_*y*_ = 64 m/s (*θ*_*SkH*_ ≈ 65°), Fig. 4(a). Besides the stretching and deformation of the skyrmionium shell, under the action of the large current the skyrmionium core is compressed. At the certain critical current this compression can lead to the core’s annihilation resulting in the transformation of the skyrmionium into the skyrmion^33^. Thus, there is the certain range of the working currents at which the skyrmionium is stable.

In the previous section, we showed the range of the magnetic parameters *K* and *D*, at which a stable skyrmionium can be realized. Now it is necessary to investigate the stability of the skyrmionium under the action of the spin current. For the found stability parameters *K* and *D* in accordance with Fig. 2(d), simulations of the skyrmionium motion under the action of the spin current for 5 ns were carried out. As a result, parameters at which the skyrmionium remains stable were found and the velocity of its motion was determined. The obtained diagrams in the coordinates *K* and *D* are shown in Fig. 4(c–e), where the color denotes the velocity of the skyrmionium motion (*V*). Comparing the data from the diagrams depicted in Figs 2(d) and 4(c–e), we can conclude that a skyrmionium of a larger radius will have the maximum velocity under the action of the same current. In this case, the small-radius skyrmioniums will annihilate under the action of the spin current resulting in the stability region narrowing. With the rising current density, the velocity increases linearly, but this also leads to a decrease in the stability region.

## Conclusion

The processes of nucleation and motion of the skyrmionium under the action of the spin polarized current in nanotracks, as well as the influence of magnetic parameters on the stability of this quasi-particle, were studied by means of micromagnetic simulations. We have determined values of the PMA energy and iDMI constant, at which the stable skyrmionium can be observed. The dependence of the skyrmionium size on these parameters was found. Zero total topological charge of the skyrmionium makes it possible to completely compensate the skyrmion Hall effect and to realize a rectilinear motion along the nanotracks under the action of the spin current. In this case, the longitudinal velocity of the skyrmionium’s motion is higher than that of the free skyrmion. The influence of the magnitude of the spin current density on the skyrmionium stability region was also investigated. The obtained results will make it possible to select the magnetic parameters and operating modes for the experimental implementation of the fast racetrack memory based on skyrmioniums.

The advanced method for the skyrmionium nucleation in a nanotrack composed a heavy metal nanoring with the help of a local enhancement of the spin Hall effect has been proposed. The results of modeling the Pt/Co/Ta system confirm the feasibility of this proof of concept. The generation of a skyrmionium occurs due to a local increase of the amplitude of the spin current and does not require the presence of a third electrical contact. This greatly simplifies the fabrication process, and also makes the proposed method more promising for a practical application and designing the skyrmionium-based memory. The creation of logic devices operating by topological charges of spin textures is also promising, but it requires additional studies to deeply understand the skyrmionium motion depending on the complex geometry of logical elements.

## Electronic supplementary material

Supplementary Movie

Supplementary File
