# Supplementary material for: Skyrmionium – high velocity without the skyrmion Hall effect
Source: Sci Rep. 2018 Nov 16;8:16966. doi: 10.1038/s41598-018-34934-2 (PMC6240074; doi:10.1038/s41598-018-34934-2)
Supplement: Supplementary file 2 — Supplementary File [file 41598_2018_34934_MOESM2_ESM.pdf]

Supplementary File

Skyrmionium – high velocity without the skyrmion Hall effect

Kolesnikov A.G.[1,*], Stebliy M.E.[1], Samardak A.S.[1,2,*], Ognev A.V.[1]

[1]*Laboratory of Thin Film Technologies, School of Natural Sciences, FEFU, Vladivostok, Russia*

[2]*National Research South Ural State University, Chelyabinsk, Russia*

e-mail addresses: *kolesnickov.a@gmail.com* (Kolesnikov A.G.), *samardak.as@dvfu.ru* (Samardak A.S.)

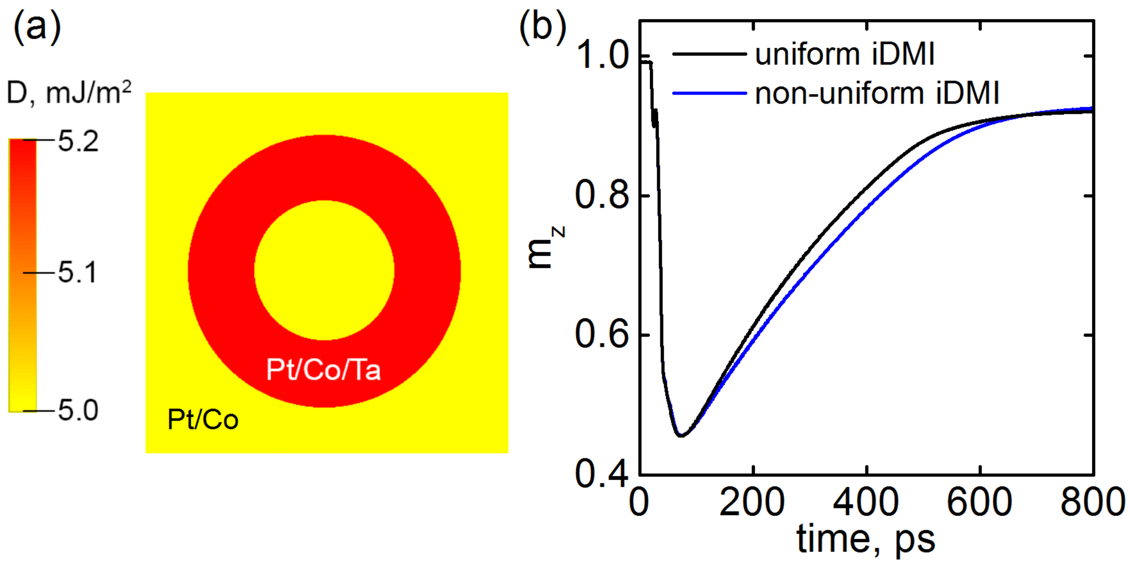

Fig.S1. *(a) The iDMI distribution map in the ferromagnetic layer. (b) Variation of the* m_z *component of the magnetization during the nucleation of a skyrmionium in cases of the uniform and non-uniform distributions of iDMI.*

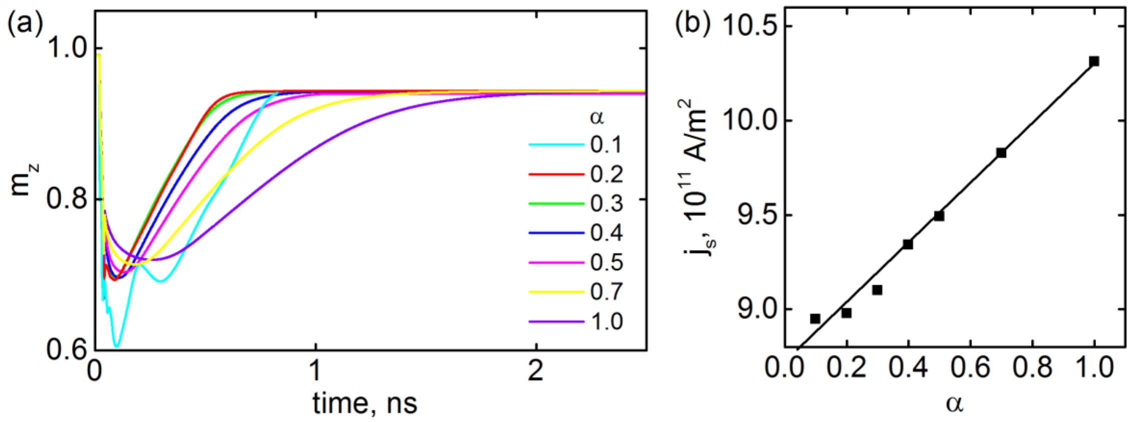

Fig.S2. *(a) Time-dependent variation of* m_z *during the skyrmionium nucleation for different values of* α*. (b) Linear dependence of the spin current magnitude on* α*.*

The **Supplementary Movie** demonstrates the skyrmionium spin dynamics under action of the spin-orbit torque as shown in Fig.1(e) of the main text.
